# Global, regional and national burden of non-Hodgkin lymphoma from 1990 to 2017: estimates from global burden of disease study in 2017

**DOI:** 10.1080/07853890.2022.2039957

**Published:** 2022-02-23

**Authors:** Haifeng Sun, Li Xue, Yahuan Guo, Jianqiang Du, Kejun Nan, Ming Li

**Affiliations:** a Department of Oncology, The First Affiliated Hospital of Xi'an Jiaotong University, Xi'an, P.R. China; b The Third Department of Medical Oncology, Shaanxi Provincial Cancer Hospital Affiliated to Medical College of Xi’an Jiaotong University, Xi'an, P. R. China; c Department of Clinical Laboratory, The Second Affiliated Hospital of Xi'an Jiaotong University, Xi'an, P. R. China; d The First Department of Medical Oncology, Shaanxi Provincial Cancer Hospital Affiliated to Medical College of Xi’an Jiaotong University, Xi'an, P. R. China; e Key Laboratory of Biomedical Information Engineering of Education Ministry, School of Life Science and Technology, Xi’an Jiaotong University, Xi’an, P. R. China; g Department of Cardiovascular Surgery, The First Affiliated Hospital of Xi'an Jiaotong University, Xi'an, P. R. China

**Keywords:** Non-Hodgkin lymphoma, incidence, deaths, EAPC

## Abstract

**Backgroud:**

Non-Hodgkin lymphoma (NHL) is a common B/NK/T cell lymphoma. We collected detailed data about the incidence and mortality of NHL from Global Burden of Disease (GBD) Study in 2017 and extensively assessed the disease burden of NHL at the global level and also analysed its current trends according to sex, age, socio-demographic index (SDI), country and region.

**Methods:**

By obtaining relevant data from Global Burden of Disease Study in 2017, estimated annual percentage changes (EAPCs) of age-standardized rate (ASR) were calculated to assess the current trends of the rate of incidence and mortality.

**Results:**

Globally, ASR of incidence in NHL was increased while ASR of mortality and its annual percentage change was relatively stable. EAPCs in the incidence of NHL decreased in the low SDI regions but increased in the high SDI regions. The ratio of male to female mortalities was the highest in the 50–69-year-old age group, especially in the middle and middle-high SDI regions.

**Conclusion:**

The incidence of NHL was increased globally, whereas the deaths and its annual percentage change were relatively stable from 1990 to 2017.

**Key messages**
- Age-standardized rate (ASR) of incidence in NHL was increased globally from 1990 to 2017.
- ASR of mortality and its annual percentage change in NHL were relatively stable globally from 1990 to 2017.
- Estimated annual percentage changes (EAPCs) in the incidence of NHL decreased in the low socio-demographic index (SDI) regions but increased in the high SDI regions.

## Introduction

Non-Hodgkin lymphoma (NHL) is a common B/NK/T cell lymphoma with 509,590 new cases and 248,724 deaths around the world in 2018 [1]. According to the report from National Central Cancer Registry of China, there were 88,200 new cases of lymphoma and myeloma in 2015 which accounted for 2.1% of all new cancer cases, whereas the case number of deaths from lymphoma and myeloma was 52,100 which accounted for 1.9% of all cancer deaths in 2015 [2]. In 2016, more than 260,000 people were diagnosed with lymphoma in China, indicating that there were approximately 20 patients with lymphoma per 100,000 people [3].

The increase in overall survival rates of NHL was largely ascribed to the progress in several treatment studies and the application of the relevant research achievements [4–9], which included monoclonal antibody (mAbs) [10–12], mAbs linked to anti-tubulin or DNA damaging agents [13], small molecule inhibitors [14,15] and the targeted chimeric antigen receptor T cells (CAR-T) [16–18]. However, recently the mortalities of NHL are still high and even remain increasing in some regions. And the distribution trend of incidence and mortality of NHL varies depending on age, gender and country. Although several regional and national studies on the incidence and mortality of NHL have been performed, specific studies about the burden of NHL at a global level are scarce.

The aim of this study was to comprehensively analyse the distribution trends of NHL in different countries and regions by collecting data from Global Burden of Disease (GBD) study. It is necessary to get more detailed data on the incidence and mortality of NHL and the changing trends of NHL in different countries and regions, which could help policymakers to adopt policies more rationally based on the information. In GBD Study in 2017, countries and regions were divided into five major regions according to the social-demographic index (SDI). Therefore in this study, we collected detailed data about the incidence and mortality of NHL from GBD Study in 2017 and comprehensively assessed the disease burden of NHL at the global level and also analysed its current trends according to sex, age, SDI, country and region. We also collected data on human development index (HDI) of the national level in 2017 from the World Bank and evaluated the association between percentage change of incidence and mortality and HDI at the national level.

## Materials and methods

### Study data

We collected data from the Global Health Data Exchange (GHDx) on GBD Study in 2017 hosted by Institute for Health Metrics and Evaluation at Washington University, with the query tool (http://ghdx.healthdata.org/gbd-results-tool). Instruments for the tabulation and graphical visualization of the GLOBOCAN database for 195 countries can be found on the Global Cancer Observatory website. The 195 countries were divided into 21 regions, including Eastern, Western, South-Eastern, and South Central Asia; Eastern, Western, Southern, and Northern Europe; Northern and South Central America; Southern, Northern, Central, Eastern, and Western Africa; Oceanian; Australia/New Zealandand Caribbean [1]. In GBD Study in 2017, countries and regions were divided into five major regions according to SDI as follows: low SDI, low-middle SDI, middle SDI, middle-high SDI and high SDI. SDI was calculated based on per capital income at the national level, the average educational years of people above 15 years old and the general fertility rate. General methods for GBD 2017 and the method for estimation of NHL burden have been detailed in other GBD studies [19,20]. Briefly, data on the incidence and mortality of NHL were sought from individual cancer registries or aggregated databases of cancer registries. Since NHL is a fatal disease, we used incidence and mortality to assess its burden. The subjects in this study were patients with NHL which conform to an approved standard of The International Classification of Diseases such as 9th/10th revision codes pertaining to NHL (200–200.9, 202–202.98 and C82–C85.29, C85.7–C86.6, C96–C96.9, respectively).

### Statistical analysis

The indices relating to age-standardized rate (ASR) are good indicators of changes in disease patterns of population distribution, as well as are clues of changes in risk factors. ASR is essential when we compare different groups of people with distinct age structures, so ASR of incidence and mortality was calculated to quantify the burden of NHL in this study. The ASR per 100,000 people was calculated using the method reported by previous study [21]. In briefly, the age-specific rates and number of people in the same age subgroup of the standard people group were sum up, then be divided by the sum of standard population weights.

The change trends of incidence and mortality of NHL were assessed by percentage changes and estimated annual percentage changes (EAPCs). EAPC is a frequently used measure of the ASR trend over a time interval. A regression line was fitted to the natural logarithm of the ASR, i.e. y = a + Px + £, where y is the natural logarithm of the ASR, and x is the calendar year. The EAPC was calculated as 100 × (exp (P) −1), and its 95% confidence intervals (CIs) can also be obtained from the model.

Additionally, in order to explore the influential factor for incidence and mortality of NHL, we collected data on HDI at the national level in 2017 from the World Bank and analysed the correlation between percentage change of incidence and mortality and HDI at the national level.

All the statistics were performed with the R program (Version 3.5.3, R core team). A *p* value of less than 0.05 was considered statistically significant.

## Results

### Global burden of non-Hodgkin lymphoma

**Figure 1.**
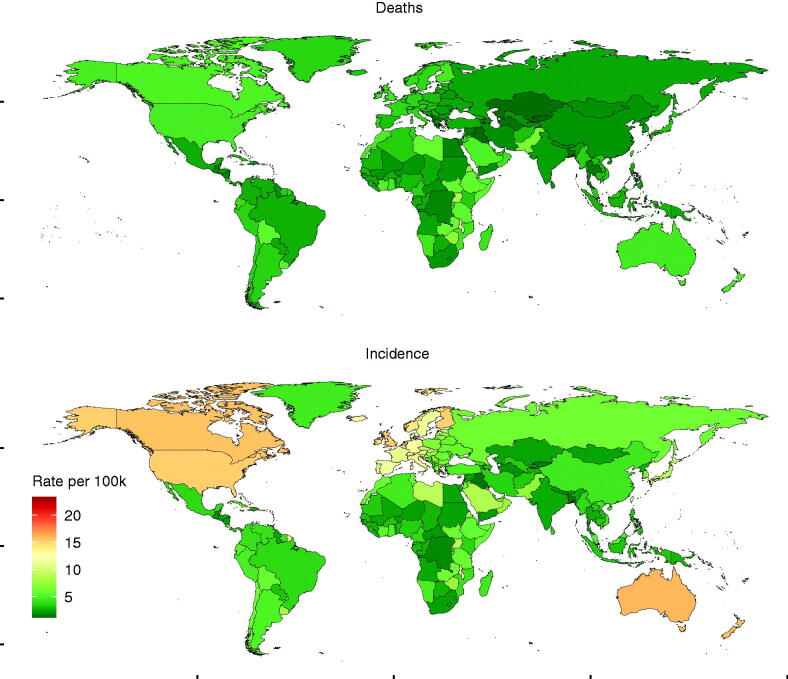
The global disease burden of non-Hodgkin lymphoma for both sexes in 195 countries and territories in 2017. The age-standardized deaths rate (upper) and the age-standardized incidence rate (lower). The world map was created using R program package “maps” (version 3.3.0, https://cran.r-project.org/web/packages/maps/index.html).

**Figure 2.**
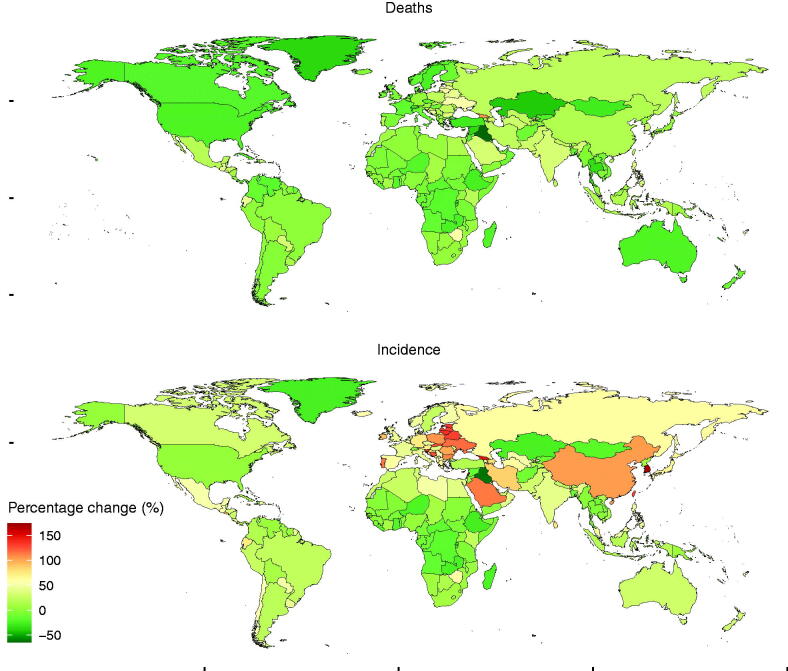
The age-standardized percentage change of incidence and mortality of non-Hodgkin lymphoma for both sexes in 195 countries and territories in 2017. The age-standardized percentage change of deaths (upper) and the age-standardized percentage change of incidence (lower). The world map was created using R program package “maps” (version 3.3.0, https://cran.r-project.org/web/packages/maps/index.html).

Globally, the incidence of NHL varies considerably across the world, with the highest incidence observed in Lebanon (23.35 per 100,000 people; 95% UI: [20.19–27.08] in 2017), followed by Australia (15.95 per 100,000 people; 95% UI: [14.1–17.89]) and New Zealand (15.73 per 100,000 people; 95% UI: [14.117.89]). The lowest incidence was observed in Iraq (1.49 per 100,000 people, 95% UI: [1.35–1.64] in 2017), followed by Kyrgyzstan (1.68 per 100,000 people; 95% UI [1.51–1.84]) and Bangladesh (1.71 per 100,000 people; 95% UI: [1.42–2.01]) (Figure 1 and Table S1). As for the percentage change of incidence, Lebanon had witnessed more than one time of increment (174.03%) from 1990 to 2017, followed by South Korea (169.07%) and Georgia (147.17%). From 1990 to 2017, Iraq had fallen (−61.35%), followed by Burundi (−33.34%) and Rwanda (−31.25%) (Figure 2 and Table S2).

As for the mortality of NHL across the world, it was shown that the mortality in Malawi was the highest (11.79 per 100,000 people; 95% UI: [9.65–14.02] in 2017), followed by Lebanon (9.02 per 100,000 people; 95% UI: [8.01–10.22]) and Uganda (8.24 per 100,000 people; 95% UI: [6.95–9.68]). Besides, the lowest mortality was observed in Kyrgyzstan (1.22 per 100,000 people; 95% UI: [1.12–1.33] in 2017), followed by Iraq (1.26 per 100,000 people; 95% UI: [1.14–1.38]) and Kazakhstan (1.41 per 100,000 people; 95% UI: [1.3–1.55]) (Figure 1 and Table S3). In terms of the percentage change of death from 1990 to 2017, more increments was recorded in Georgia (104.74%), followed by Lithuania (59.94%) and Zimbabwe (59.22%), more reductions were recorded in Iraq (−64.62%), followed by Kazakhstan (−42.17%) and Bermuda (−41.44%) (Figure 2 and Table S4).

**Figure 3.**
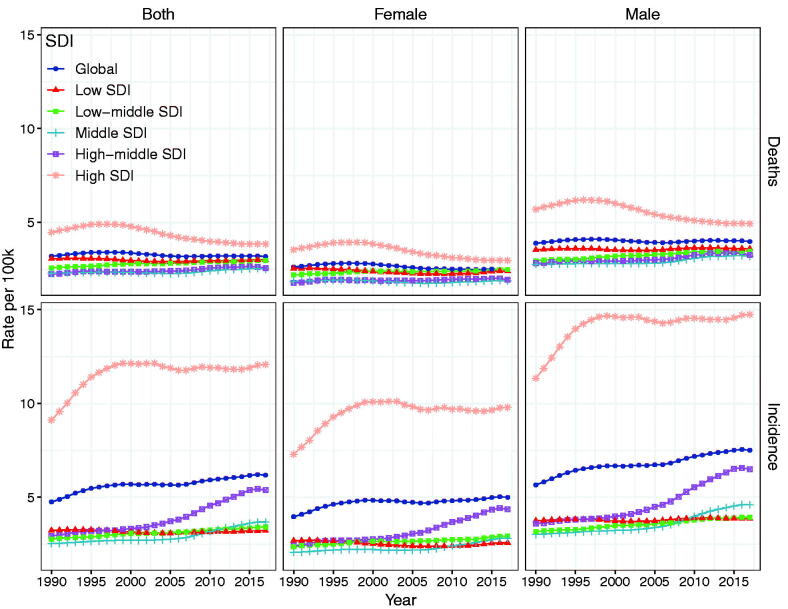
The age-standardized incidence and mortality rate of non-Hodgkin lymphoma by SDI and gender from 1990 to 2017.

Regarding SDI regions, the incidence of NHL was increased in other SDI regions except the low SDI regions during the period from 1990 to 2017 (Table 1). As for the geographical regions, absolute number of NHL cases was increased in almost all the regions (Figure 3). Significant or slight decreases in the percentage change incidence of NHL were reported in 50 countries or territories (including Iraq, Burundi, Rwanda, etc.) from 1990 to 2017. However, the percentage change incidence of NHL was reported as being significantly increased in 18 countries or territories including Lebanon, South Korea, Georgia, Estonia, etc. Additionally, 33 countries or territories including Seychelles, Romania, Mauritius, and Ireland showed a substantial increase. But steady or slight increase in the percentage change incidence of NHL was reported in 93 countries or territories, including France, UK, Philippines, Norway, North Korea, etc (Figure 2).

**Table 1.** The age-standardized rate of incidence and death of non-Hodgkin lymphoma in 1990 and 2017, and age-standardized percentage change and EAPC of non-Hodgkin lymphoma from 1990 to 2017.

Location	Death (1990, 95% UI)	Death (2017, 95% UI)	Incidence (1990, 95% UI)	Incidence (2017, 95% UI)	Percentage change death (1990–2017, 95% UI)	Percentage change incidence (1990–2017, 95% UI)	EAPC (death, 95% CI)	EAPC (incidence, 95% CI)
Global	3.19 (3.1 to 3.34)	3.18 (3.11 to 3.24)	4.75 (4.62 to 4.93)	6.18 (6.06 to 6.29)	−0.47% (−5.91 to 3.34)	30.12% (23.92 to 35.29)*	−0.19 (−0.28 to −0.1)*	0.75 (0.62 to 0.88)*
Low SDI	3.05 (2.59 to 3.76)	2.99 (2.66 to 3.21)	3.23 (2.69 to 4.06)	3.22 (2.86 to 3.46)	−2.03% (−19.39 to 16.38)	−0.55% (−20.15 to 21.92)	−0.15 (−0.23 to −0.08)*	−0.1 (−0.19 to −0.02)*
Low-middle SDI	2.57 (2.39 to 2.81)	2.96 (2.74 to 3.17)	2.76 (2.55 to 3.06)	3.41 (3.16 to 3.67)	15.13% (0.23 to 26.02)*	23.42% (5.92 to 36.03)*	0.5 (0.46 to 0.54)*	0.74 (0.71 to 0.78)*
Middle SDI	2.29 (2.2 to 2.37)	2.51 (2.42 to 2.61)	2.52 (2.43 to 2.61)	3.68 (3.54 to 3.83)	9.62% (3.86 to 17.01)*	45.61% (37.23 to 55.26)*	0.32 (0.21 to 0.43)*	1.4 (1.19 to 1.61)*
High-middle SDI	2.24 (2.16 to 2.32)	2.56 (2.49 to 2.64)	2.94 (2.84 to 3.05)	5.38 (5.19 to 5.56)	14.16% (7.96 to 20.82)*	82.91% (71.85 to 93.49)*	0.54 (0.45 to 0.63)*	2.46 (2.21 to 2.7)*
High SDI	4.47 (4.43 to 4.5)	3.84 (3.75 to 3.92)	9.11 (9.01 to 9.21)	12.07 (11.75 to 12.39)	−14.04% (−15.99 to −12.17)*	32.51% (28.71 to 36.27)*	−0.99 (−1.18 to −0.8)*	0.58 (0.32 to 0.85)*
Central Europe	2.56 (2.52 to 2.6)	2.88 (2.8 to 2.97)	3.86 (3.78 to 3.95)	7.1 (6.84 to 7.4)	12.46% (8.56 to 16.37)*	83.91% (75.73 to 92.5)*	0.51 (0.36 to 0.65)*	2.5 (2.39 to 2.61)*
Australasia	5.85 (5.72 to 5.99)	4.6 (4.2 to 4.99)	12.35 (11.96 to 12.75)	15.91 (14.31 to 17.54)	−21.46% (−28.37 to −14.64)*	28.85% (15.48 to 42.95)*	−1.46 (−1.69 to −1.24)*	0.55 (0.33 to 0.77)*
Central Asia	1.86 (1.7 to 1.98)	1.88 (1.79 to 1.97)	2.41 (2.22 to 2.55)	2.98 (2.83 to 3.14)	1.46% (−6.92 to 12.69)	23.82% (13.68 to 36.56)*	−0.09 (−0.2 to 0.02)	0.69 (0.5 to 0.88)*
Central Latin America	2.57 (2.52 to 2.61)	2.66 (2.55 to 2.78)	2.87 (2.81 to 2.93)	3.82 (3.66 to 4.03)	3.78% (−0.84 to 9.38)	33.42% (27.33 to 40.66)*	0.09 (0.01 to 0.16)*	1.04 (0.96 to 1.12)*
Tropical Latin America	2.71 (2.65 to 2.77)	2.74 (2.66 to 2.82)	3.05 (2.97 to 3.12)	3.77 (3.65 to 3.89)	1.11% (−2.58 to 4.84)	23.47% (18.7 to 28.08)*	−0.16 (−0.42 to 0.1)	0.62 (0.36 to 0.88)*
Caribbean	4.01 (3.8 to 4.51)	3.45 (3.2 to 3.79)	4.92 (4.62 to 5.54)	5.35 (4.96 to 5.83)	−14.08% (−19.91 to −8.3)*	8.84% (0.43 to 17.15)*	−0.62 (−0.79 to −0.46)*	0.3 (0.13 to 0.47)*
Southern Sub-Saharan Africa	2.61 (2.35 to 2.9)	2.99 (2.78 to 3.19)	2.7 (2.45 to 2.97)	3.25 (3.01 to 3.47)	14.82% (3.88 to 27.99)*	20.52% (10.18 to 33.53)*	0.63 (0.15 to1.11)*	0.76 (0.28 to 1.24)*
Eastern Europe	2 (1.84 to 2.1)	2.57 (2.51 to 2.64)	3.5 (3.22 to 3.73)	6.14 (5.84 to 6.46)	28.62% (23.06 to 38.32)*	75.3% (63.01 to 90.61)*	1.18 (0.97 to 1.38)*	2.6 (2.2 to 3)*
Southern Latin America	3.67 (3.58 to 3.77)	3.62 (3.35 to 3.93)	4.21 (4.1 to 4.32)	5.48 (5.08 to 5.97)	−1.52% (−8.93 to 7.12)	30.29% (20.53 to 41.76)*	−0.29 (−0.59 to 0.01)	0.76 (0.51 to 1.02)*
Andean Latin America	3.63 (3.38 to 3.9)	4.02 (3.65 to 4.37)	3.86 (3.61 to 4.16)	5.09 (4.62 to 5.56)	10.73% (−1.11 to 23.24)	31.84% (16.83 to 47.17)*	0.39 (0.16 to 0.63)*	1.08 (0.87 to 1.3)*
Southeast Asia	2.79 (2.58 to 3.04)	2.78 (2.61 to 2.94)	3 (2.76 to 3.29)	3.46 (3.24 to 3.67)	−0.41% (−9.26 to 9.33)	15.1% (3.66 to 26.49)*	−0.06 (−0.13 to 0.01)	0.46 (0.41 to 0.5)*
Western Europe	4.18 (4.13 to 4.22)	3.77 (3.63 to 3.91)	8.4 (8.23 to 8.56)	12.67 (12.11 to 13.24)	−9.63% (−13.13 to −6.35)*	50.95% (43 to 58.65)*	−0.79 (−0.99 to −0.58)*	1.15 (0.87 to 1.42)*
High-income Asia Pacific	3.09 (3.05 to 3.13)	3.18 (3.02 to 3.31)	5.08 (4.97 to 5.19)	8.61 (7.99 to 9.22)	2.89% (−2.09 to 7.55)	69.38% (57.2 to 81.34)*	−0.08 (−0.24 to 0.08)	2.09 (1.93 to 2.25)*
South Asia	2.13 (1.94 to 2.36)	2.68 (2.5 to 2.83)	2.22 (2.02 to 2.48)	2.96 (2.76 to 3.13)	25.69% (10.35 to 39.58)*	33.12% (15.42 to 47.55)*	0.85 (0.79 to 0.9)*	1.05 (0.98 to 1.12)*
High-income North America	6.2 (6.13 to 6.27)	4.74 (4.62 to 4.87)	14.37 (14.12 to 14.61)	15.24 (14.77 to 15.75)	−23.51% (−25.71 to −21.17)*	6.05% (2.23 to 10.12)*	−1.58 (−1.81 to −1.35)*	−0.52 (−0.88 to −0.15)*
East Asia	1.92 (1.82 to 2.01)	2.21 (2.12 to 2.31)	2.24 (2.12 to 2.34)	4.55 (4.32 to 4.76)	15.21% (6.99 to 24.96)*	103.37% (87.02 to 120.8)*	0.57 (0.25 to 0.9)*	2.8 (2.27 to 3.33)*
North Africa and Middle East	3.05 (2.76 to 3.46)	2.81 (2.67 to 2.99)	3.33 (3.03 to 3.82)	4.34 (4.12 to 4.63)	−7.63% (−21.32 to 6.51)	30.29% (9.24 to 49.14)*	−0.25 (−0.34 to −0.16)*	1.11 (1.06 to 1.17)*
Oceania	2.59 (2.14 to 3.37)	2.77 (2.31 to 3.66)	2.81 (2.35 to 3.72)	3.11 (2.63 to 4.16)	7.27% (−5.35 to 21.8)	10.49% (−3.06 to 27.45)	0.41 (0.33 to 0.49)*	0.51 (0.44 to 0.58)*
Central Sub-Saharan Africa	2.49 (1.84 to 2.96)	2.08 (1.47 to 2.67)	2.56 (1.95 to 3.07)	2.16 (1.57 to 2.71)	−16.46% (−32.18 to 2.34)	−15.9% (−32.99 to 5.12)	−0.75 (−0.92 to −0.58)*	−0.73 (−0.9 to −0.57)*
Eastern Sub-Saharan Africa	6.57 (5.48 to 8.12)	5.63 (4.84 to 6.27)	6.9 (5.62 to 8.71)	5.96 (5.1 to 6.67)	−14.29% (−34.81 to 9.76)	−13.58% (−35.87 to 14.57)	−0.79 (−0.9 to −0.68)*	−0.76 (−0.87 to −0.64)*
Western Sub-Saharan Africa	3.26 (2.76 to 3.91)	3.17 (2.79 to 3.68)	3.6 (3 to 4.34)	3.56 (3.11 to 4.13)	−2.57% (−20.26 to 16.92)	−1.29% (−19.8 to 20.08)	−0.29 (−.41 to −0.18)*	−0.27 (−0.4 to −0.13)*

Results are for both sexes combined. *Statistically significant increase or decrease.

The mortality was increased in the low-middle, middle and middle-high SDI regions during the period from 1990 to 2017. However, the mortality was reduced in the low and high SDI regions (Table 1). One hundred countries or territories (such as Iraq, Kazakhstan, Bermuda, etc.) were shown as being significant or slight decreases in the percentage change death of NHL from 1990 to 2017. Moeover, substantial increase in the percentage change death was reported in five countries or territories including Lithuania, Zimbabwe, and Ukraine. Steady or slight increase in the percentage change death was shown in 89 countries or territories including Japan, South Africa, India, etc. (Figure 2).

### The change in the incidence of NHL

At the global level, the incidence of NHL increased gradually, and it changed from 4.75 per 100,000 people (95% UI: 4.62–4.93) in 1990 to 6.18 per 100,000 (95% UI: 6.06–6.29) in 2017 (Figure 3). The percentage change incidence of NHL was 30.12%, while its EAPCs of the incidence was 0.75 (95% CI: 0.62–0.88) during the period from 1990 to 2017 (Table 1). In terms of SDI level, as shown in Figure 3 and Table 1, the percentage change incidence and its EAPCs of the incidence decreased slightly in the low SDI region but they increased significantly in the other SDI regions. In the high-middle SDI region, EAPCs of the incidence increased dramatically by 2.46 (95% CI: 2.21 to − 2.7). EAPCs of the incidence in the other three SDI regions were on the increase.

**Figure 4.**
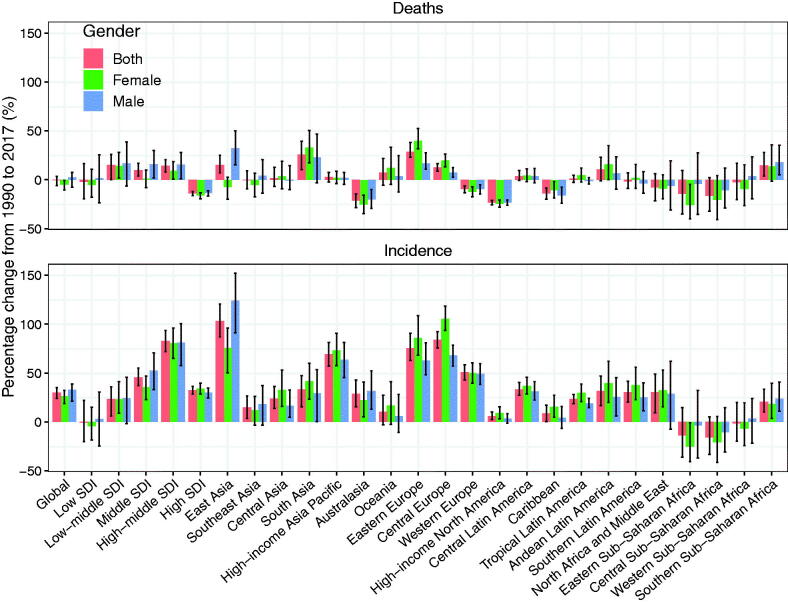
The age-standardized percentage change of incidence and mortality of non-Hodgkin lymphoma by SDI and gender from 1990 to 2017.

**Figure 5.**
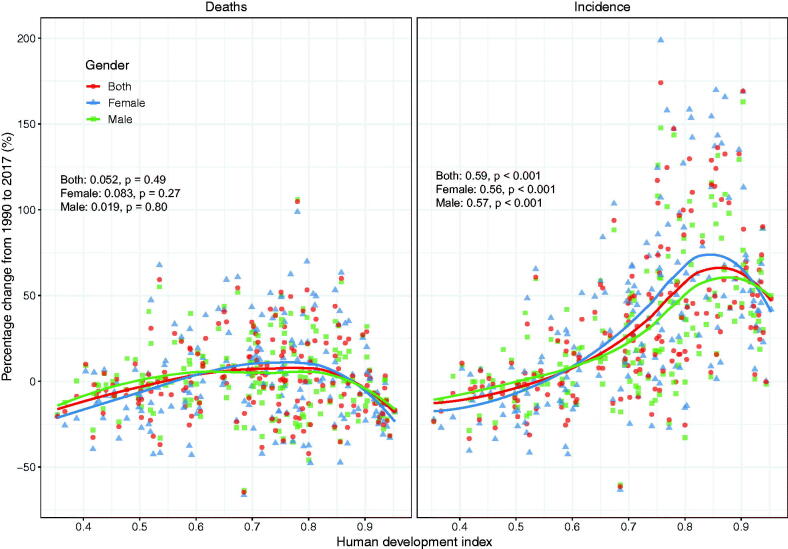
The correlation between human development index (HDI) and age-standerdized percentage change of incidence and mortality of non-Hodgkin lymphoma from 1990 to 2017. The dots represent countries that were available on HDI data. The p indices and *p* values were derived from Pearson correlation analysis.

From the viewpoint of the 21 territories in the world, the percentage change incidence of NHL in East Asia, High-income Asia Pacific, Eastern Europe and Central Europe increased significantly from 1990 to 2017. However, the percentage change incidence decreased in the following three territories such as Central, Eastern and Western sub-Saharan Africa (Figure 4). Besides, we observed positive correlations between HDI and incidence of NHL (*ρ* = 0.59, *p* < 0.001) for both males (*ρ* = 0.57, *p* < 0.001) and females (*ρ* = 0.56, *p* < 0.001) (Figure 5).

**Figure 6.**
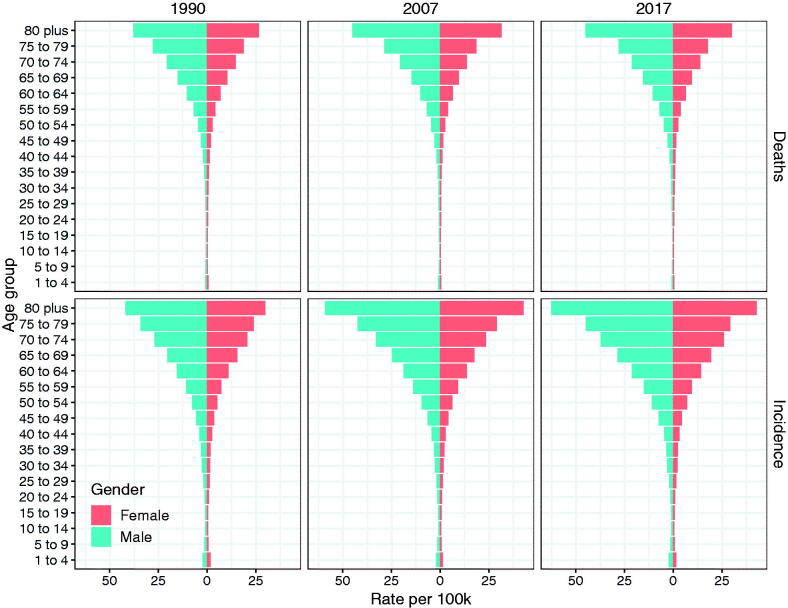
The incidence and mortality of non-Hodgkin lymphoma in different age groups in 1990, 2007 and 2017.

**Figure 7.**
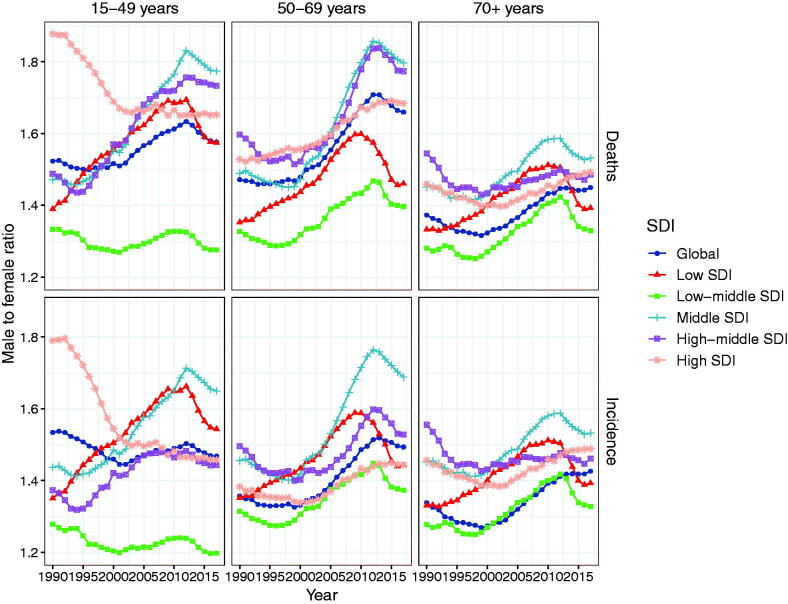
The male to female ratio of incidence and mortality of non-Hodgkin lymphoma among different age groups from 1990 to 2017.

As for gender and age, the incidence of NHL in the males was higher than that in the females (Figure 6). For example, in 2017, the ASR of NHL incidence was 46.85 (95% UI: 45.29–48.44) and 32.86 (95% UI 31.67–33.89) per 100,000 people in males and females aged over 70, respectively. The ASR of NHL incidence was 17.54 (95% UI: 16.98–18.07) and 11.75 (95% UI: 11.35–12.12) per 100,000 people in males and females aged 50–69, respectively. The ASR of NHL incidence was 3.14 (95% UI: 3.02–3.25) and 2.14 (95% UI: 2.07–2.22) per 100,000 people in males and females aged 15–49, respectively. Globally, the male-to-female ratios of incidence were all more than 1.4 in the groups aged 15–49, 50–69 and over 70. Besides, the male-to-female ratios of incidence were greater in Middle SDI regions of all three age groups than those in low-middle SDI regions of all three age groups (Figure 7). Noticeably, the male-to-female ratio of incidence in high-middle SDI region had been increasing significantly since 2005 (Figure 7). In brief, the incidence of NHL in the young-aged group accounted for relatively low proportion in all the age groups, while the incidence of NHL was significantly higher in the males of old-aged groups than that in the females of old-aged groups (Figure 6).

Overall, the male-to-female ratio of incidence tended to be on the increase from 1990 to 2017. However, there were also exceptions. In the group aged 15–49, the male-to-female ratio of incidence in high SDI regions gradually decreased, and that in low SDI regions were relatively stable. The male-to-female ratio of incidence in low-middle SDI regions of all three age groups was lower than those in the other four SDI regions of all three age groups. As regard to the level of GBD territories, the incidence rate of NHL showed an upward trend in four territories including East Asia, High-income Asia Pacific, Eastern Europe, and Central Europe, while a downward trend was observed in three territories including Central, Eastern, and Western sub-Saharan Africa.

### The change in the mortality of NHL

At the global level, the mortality of NHL was stable with 3.19 per 100,000 people (95% UI: 3.1–3.34) in 1990 and 3.18 per 100,000 people (95% UI: 3.11–3.24) in 2017. However, the EAPCs of NHL significantly decreased by −0.19 (95% CI: from −0.28 to −0.1), and the percentage change mortality of NHL from 1990 to 2017 decreased by −0.47% (95% UI: from −5.91 to −3.34) (Table 1). As for the analysis of NHL according to the SDI level and territories, the percentage change death in both low and high SDI regions decreased significantly during the period from 1990 to 2017, and its percentage change death was −2.03% (95% UI: from −19.39 to 16.38) and −14.04% (95% UI from −15.99 to −12.17) in low and high SDI, respectively (Table 1). From 1990 to 2017, EAPCs of death in low and High SDI regions decreased slightly with −0.15 (95% CI: from −0.23 to −0.08) and − 0.99(95% CI: from −1.18 to −0.8), respectively. But the percentage change death of NHL and its EAPCs in other SDI regions increased from 1990 to 2017 (Table 1). The mortality in males was higher than that in the females during this period (Figure 3). Moreover, the percentage change death of NHL and its EAPC were reported as being slightly decreased in 10 territories including Australasia, Caribbean, Southern Latin America, Southeast Asia, Western Europe, High-income North America, North Africa and the Middle East, Central Sub-Saharan Africa, Eastern Sub-Saharan Africa and Western Sub-Saharan Africa. And the percentage change death of NHL and its EAPC were shown as being steadily or slightly increased in eleven territories including Central Europe, Central Asia, Central Latin America, Tropical Latin America, Southern Sub-Saharan Africa, Eastern Europe, Andean Latin America, High-income Asia Pacific, South Asia, East Asia and Oceania (Table 1). From the viewpoint of GBD territories, the mortality showed an upward trend in six territories (East Asia, South Asia, Eastern Europe, Central Europe, Andean Latin American, Southern Sub-Saharan Africa), a downward trend in eight territories (Australasia, High-income North American, Western Europe, Caribbean, North Africa and Middle East, Central Eastern and Western sub-Saharan Africa), and a stable trend in the other territories (Figure 4).

As for gender and age, the mortality of NHL had been gradually increasing with age since 1990, and the mortality of the males was higher than that of the females (Figure 6). For example, in 2017, the mortality of NHL in patients aged over 70 was 30.12 (95% UI: 29.32–30.83) and 20.78 (95% UI: 20.12–21.35) per 100,000 people for males and females, respectively. The mortality in patients aged 50–69 was 8.56 (95% UI: 8.29–8.78) and 5.16 (95% UI: 4.98 − 5.34) per 100, 000 people for the males and the females, respectively. The mortality of people aged 15–49 was 1.24 (95% UI: 1.19–1.29) and 0.79 (95% UI: 0.76 − 0.83) per 100,000 for the males and females, respectively. The male-to-female ratio of deaths displayed an increasing tendency in the age groups of 15–49, 50–69 and over 70 years from 1990 to 2017. However, in the group aged 15–49, the male-to-female ratios of deaths in high SDI regions gradually decreased.

In brief, the male-to-female ratios of deaths in the group aged over 70 were lower than that in the groups aged 15–49 and 50–69. Additionally, the mortality of NHL in the young-aged group accounted for relatively lower proportion in all the aged groups. And for the old-aged group, males showed a significantly higher mortality of NHL as compared to females. Moreover, the male-to-female ratios of deaths in Middle SDI regions were relatively higher than that in other regions in all three age groups. But low-middle SDI region showed a lower male-to-female ratio of deaths relative to other regions in all three age groups (Figure 7).

## Discussion

In the current study, we comprehensively analysed the current trends in the incidence and mortality of NHL at the global, regional, and national level. In general, both the incidence and mortality of NHL were increased during the period from 1990 to 2017. The trend, however, varied from region to region. On the basis of the objective evidence provided in this study, more rational studies on strategies, disease control and prevention may be conducted.

NHL is a heterogeneous group of lymphoid malignancies originating in B-lymphocytes, T-lymphocytes, or natural killer (NK) cells (NK/T-cell lymphomas are very rare). According to the classifications of NHL by World Health Organisation (WHO) in 2017, there were nearly 100 subtypes [22]. The aetiology and risk factors responsible for NHL include gene rearrangement [23], chromosome translocation [24], and viruses such as Epstein-Barr virus (EBV) [3,25], human immunodeficiency virus (HIV) [26,27], hepatitis B/C virus (HBV/HCV) [28–30], helicobacter pylori (HP) [31] and human herpes virus (HHV8) [32] infection. Therefore, the global distribution of NHL shows an inconsistent tendency. Despite an increasing understanding of the pathology and genetics of NHL, global reports on distribution characteristics of the incidence of NHL remained limited. We comprehensively analysed the temporal trends in the incidence and mortality of NHL at the global level from 1990 to 2017, so as to better formulate the management program of NHL and improve the survival rate of the disease.

Data about the incidence and mortality of diseases in many countries are available through the WHO, but the cause of deaths varied greatly in the accuracy of the record and the completeness of registrations. Overall, the incidence of NHL was high in Australia, High-income North America and Western Europe, but was low in Europe, Latin America and Africa. The heterogeneous pattern in risk factor exposures results in a markedly diverse in the incidence of NHL across the world, and makes the prevention of NHL be complex. Moreover, marked variations in incidence rates of NHL exist in population of each region around the world. So, special attention should be given to the role of endemic infections and environmental exposures in the incidence of NHL in some regions, particularly in Africa, Asia and Latin America. Additionally, the mortalities of NHL were high in Eastern Sub-Saharan Africa, High-income North America and Australia, but were low in East Asia, Central Asia and Central Sub-Saharan Africa. We found the phenomenon that the mortality of NHL was high in both the developed regions and underdeveloped areas, which may be ascribed to the so-called westernization of lifestyle, the different pathological subtype of lymphoma, and relatively high incidence of invasive NHL in both the developed and underdeveloped regions. The above analysis could provide the explanation for possible causes of the overall differences in the incidence and mortality of NHL in different regions. Next, we will analyse the percentage changes from 1990 to 2017, trends by gender, age and region, and the related factors that may have effects on these results.

In this study, we described the latest trends and patterns of global incidence, mortality and EAPC of NHL from 1990 to 2017 according to the results of GBD Study in 2017. The incidence and mortality of NHL were higher in males of all age groups than that in females of all age groups, which is partly attributed to some risk factors including smoking and infections. From the point of view of the global level, the incidence of NHL had gradually increased from 4.75 per 100,000 people in 1990 to 6.18 per 100,000 people in 2017. Over the past 28 years, the incidence and EAPCs of NHL had increased by 30.11% and 0.75, respectively. Additionally, we also found that the incidence and mortality of NHL in all the SDI regions were lower in young-aged group than those in the old-aged group. An upward trend of the incidence was shown in four territories including East Asia, High-income Asia Pacific, Eastern Europe, and Central Europe, which may be associated with the continuous advancement of diagnosis and treatment in these regions. For example, stem cell transplantation and novel agents such as small molecule inhibitors and immune checkpoint inhibitors [33] could provide a new choice for those with refractory or recurrent NHL. With the development of new treatment strategies and palliative medicine, the survival period of patients with NHL is extended. Therefore, the increased incidence may reflect the improvements in the ability of early detection of subclinical lesions. However, a downward trend of the incidence was shown in three territories including Central, Eastern and Western sub-Saharan Africa, and this may be associated with wars and condition of economy and medicine in these regions. Our results also showed that EAPCs of incidence decreased most significantly by −0.1 in the low SDI region, while it increased most markedly by 2.46 in the high SDI region. In this study, we also analysed the disease burden according to the HDI which was created by the United Nations Development Program to highlight the importance of national policy in assessing outcomes of the development beyond the economic growth. We found that HDI was positively associated with the incidence of NHL, well demonstrating our presumption of the causes for the constant increase of the incidence.

Mortality is a better parameter for evaluating the effectiveness of cancer treatment as compared to the incidence or survival rate, since mortality is less affected by some biases derived from changes in detection practices of diseases [34]. However, the advantage of mortality in assessing the efficacy of treatment is based on the premise that survival rate is steady between the groups to be compared. We found that from the viewpoint of global level, the EAPCs of death of NHL decreased significantly by −0.19 during the period from 1990 to 2017, while the percentage change of mortality of NHL decreased by −0.47 over the same period. Notably, the mortality of NHL in male subjects was higher than that in female subjects. Moreover, our results showed that the mortality of NHL was relatively stable around the world. Based on the above analysis, we should actively prevent or reduce the incidence of NHL other than providing better approaches for treatment of NHL, although there is a great challenge to complete the task.

There are some limitations in this study. First, the quality of data in some underdeveloped territories such as Africa and Tropical Latin America was not accurate, since there are not reliable systems providing the information about mortality of diseases in those regions and population-based cancer registries are rare there. Secondly, racial differences were neglected in the GBD Study. However, to a certain extent, racial differences have a profound impact on the onset and death of diseases. It is required to further investigate the aetiology and risk factors for NHL in our future study. This might help to better explain the changing trend and the distribution of pathogenic factors in the disease so as to develop more appropriate strategies of prevention and therapies of NHL.

## Supplementary Material

Supplemental Material

## Data Availability

The datasets generated during and/or analysed during the current study are available from the Global Health Data Exchange query tool (http://ghdx. healthdata.org/gbd-results-tool).

## Abbreviations

**Abbreviations**
NHL: Non-Hodgkin lymphoma
mAbs: monoclonal antibody
CAR-T: antigen receptor T cells
GBD: Global Burden of Disease
GHDx: Global Health Data Exchange
GBD: Global Burden of Disease
UIs: uncertainty intervals
ASR: age-standardized rate
CIs: confidence intervals
HDI: human development index
SDI: social-demographic index
NK cells: natural killer cells
EAPCs: Estimated annual percentage changes
DLBCL: diffuse large B-cell lymphoma
CLL: chronic lymphocytic leukaemia
SLL: small lymphocytic lymphoma
FL: follicular lymphoma
MZL: marginal zone lymphoma
MCL: mantle cell lymphoma
PTCL: peripheral T-cell lymphoma

## References

[1] Bray F, Ferlay J, Soerjomataram I, et al. Global cancer statistics 2018: GLOBOCAN estimates of incidence and mortality worldwide for 36 cancers in 185 countries. CA Cancer J Clin. 2018;68(6):394–424. 30207593 10.3322/caac.21492

[2] Chen W, Zheng R, Baade PD, et al. Cancer statistics in China, 2015. CA Cancer J Clin. 2016;66(2):115–132. 26808342 10.3322/caac.21338

[3] Pagliuca S, Bommier C, Michonneau D, et al. Epstein-Barr virus-associated post-transplantation lymphoproliferative disease in patients who received anti-CD20 after hematopoietic stem cell transplantation. Biol Blood Marrow Transplant. 2019;25(12):2490–2500. 31421238 10.1016/j.bbmt.2019.08.006

[4] Sun X, Zhen Z, Lin S, et al. Treatment outcome of chinese children with anaplastic large cell lymphoma by using a modified B-NHL-BFM-90 protocol. Pediatr Hematol Oncol. 2014;31(6):518–527. 25116372 10.3109/08880018.2014.939793

[5] Sidaway P. Haematological cancer: obinutuzumab effective against treatment-refractory NHL. Nat Rev Clin Oncol. 2016;13(8):466. 10.1038/nrclinonc.2016.112 27402577

[6] Sharma R. The burden of prostate cancer is associated with human development index: evidence from 87 countries, 1990–2016. Epma J. 2019;10(2):137–152. 31258819 10.1007/s13167-019-00169-y PMC6562055

[7] Schwarzbich MA, Schoning T, Cremer M, et al. Efficacy and toxicity of a rituximab and methotrexate based regimen (GMALL B-ALL/NHL 2002 protocol) in high risk diffuse large cell B-cell lymphoma patients as a first line treatment. Leuk Lymphoma. 2016;57(7):1723–1726. 26980422 10.3109/10428194.2015.1113274

[8] Mejia M, Perez A, Watson H, et al. Successful treatment of severe type B lactic acidosis in a patient with HIV/AIDS-associated high-grade NHL. Case Reports Immunol. 2018;2018:9093623. 30302295 10.1155/2018/9093623 PMC6158940

[9] Kuittinen T, Wiklund T, Remes K, et al. Outcome of progressive disease after autologous stem cell transplantation in patients with non-Hodgkin's lymphoma: a nation-wide survey. Eur J Haematol. 2005;75(3):199–205. 16104875 10.1111/j.1600-0609.2005.00481.x

[10] Li P, Zhao H, Ma Y, et al. A phase I, dose-escalation study of ADG106, a fully human anti-CD137 agonistic antibody, in subjects with advanced solid tumors or relapsed/refractory non-Hodgkin lymphoma. J Clin Oncol. 2020;38(15_suppl):3105–3105.

[11] Morschhauser F, Carlo-Stella C, Offner F, et al. Dual CD20-Targeted therapy with concurrent CD20-TCB and obinutuzumab shows highly promising clinical activity and manageable safety in relapsed or refractory B-cell non-Hodgkin lymphoma: preliminary results from a phase Ib trial. Blood. 2019;134(Supplement_1):1584–1584.

[12] Doyle C, Smith J, Bello C, et al. Efficacy and safety of yttrium-90 ibritumomab tiuxetan in the treatment of non-Hodgkin lymphoma. J Clin Oncol. 2020;38(5_suppl):103–103.31675248

[13] Maakaron J, Zhao Q, Puto M, et al. Phase I dose-escalation study of venetoclax plus BEAM followed by autologous stem cell transplant (ASCT) for chemoresistant or high-risk relapsed/refractory non-Hodgkin lymphoma (NHL). Blood. 2019;134(Supplement_1):2024–2024.

[14] Miles R, Galardy P. Resistance to proteasome inhibitor therapy in non-Hodgkin lymphoma. In: Xavier A, Cairo M (Eds.). Resistance to targeted therapies in lymphomas. Resistance to targeted anti-cancer therapeutics. 2019. Vol. 21. p. 71–86.

[15] Hagner P, Chiu H, Chopra V, et al. Interactome of aiolos/ikaros in diffuse large B-Cell lymphoma (DLBCL) reveals novel combination of cereblon modulators (CELMoD) and histone deacetylase (HDAC) inhibitors. Blood. 2019;134(Supplement_1):306–306.

[16] Zeng C, Cheng J, Li T, et al. Efficacy and toxicity for CD22/CD19 chimeric antigen receptor T-cell therapy in patients with relapsed/refractory aggressive B-cell lymphoma involving the gastrointestinal tract. Cytotherapy. 2020;22(3):166–171. 32063474 10.1016/j.jcyt.2020.01.008

[17] Kilgore K, Mohammadi I, Schroeder A, et al. Medicare patients receiving chimeric antigen receptor T-cell therapy for non-Hodgkin lymphoma: a real-world look at patient characteristics, healthcare utilization and costs. Biol Blood Marrow Transplant. 2020;26(3):S43–S44.

[18] George P, Dasyam N, Giunti G, et al. Third-generation anti-CD19 chimeric antigen receptor T-cells incorporating a TLR2 domain for relapsed or refractory B-cell lymphoma: a phase I clinical trial protocol (ENABLE). BMJ Open. 2020;10(2):e034629. 10.1136/bmjopen-2019-034629 PMC7044946 32041862

[19] Collaborators GBDCoD: global, regional, and national age-sex-specific mortality for 282 causes of death in 195 countries and territories, 1980-2017: a systematic analysis for the global burden of disease study 2017. Lancet. 2018;392(10159):1736–1788. 30496103 10.1016/S0140-6736(18)32203-7 PMC6227606

[20] Disease GBD, Injury I, Prevalence C. Global, regional, and national incidence, prevalence, and years lived with disability for 354 diseases and injuries for 195 countries and territories, 1990–2017: a systematic analysis for the global burden of disease study 2017. Lancet. 2018;392(10159):1789–1858. 30496104 10.1016/S0140-6736(18)32279-7 PMC6227754

[21] Zhou L, Deng Y, Li N, et al. Global, regional, and national burden of Hodgkin lymphoma from 1990 to 2017: estimates from the 2017 global burden of disease study. J Hematol Oncol. 2019;12(1):107. 31640759 10.1186/s13045-019-0799-1 PMC6805485

[22] Shankland KR, Armitage JO, Hancock BW. Non-Hodgkin lymphoma. Lancet. 2012; 380(9844):848–857. 22835603 10.1016/S0140-6736(12)60605-9

[23] Nachmias B, Sandler V, Slyusarevsky E, et al. Evaluation of cerebrospinal clonal gene rearrangement in newly diagnosed non-Hodgkin's lymphoma patients. Ann Hematol. 2019;98(11):2561–2567. 31515574 10.1007/s00277-019-03798-5

[24] Chiu BC, Blair A. Pesticides, chromosomal aberrations, and non-Hodgkin's lymphoma. J Agromed. 2009;14(2):250–255. 10.1080/10599240902773140 PMC2790329 19437285

[25] Muncunill J, Baptista MJ, Hernandez-Rodriguez A, et al. Plasma Epstein-Barr virus load as an early biomarker and prognostic factor of human immunodeficiency virus-related lymphomas. Clin Infect Dis. 2019;68(5):834–843. 29982484 10.1093/cid/ciy542

[26] Song M, Bassig B, Bender N, et al. Associations of viral seroreactivity with AIDS-related non-Hodgkin lymphoma. AIDS Res Hum Retroviruses. 2020;36(5):381–388. 31789046 10.1089/aid.2019.0208 PMC7232664

[27] Hinkle C, Makar G, Brody J, et al. HIV-associated “Double-Hit” lymphoma of the tonsil: a first reported case. Head Neck Pathol. 2020;14(4):1129–1133. 31997133 10.1007/s12105-020-01135-1 PMC7669924

[28] Zhu X, Jing L, Li X. Hepatitis C virus infection is a risk factor for non-Hodgkin lymphoma: a MOOSE-compliant meta-analysis. Medicine. 2019;98(11):e14755. 30882645 10.1097/MD.0000000000014755 PMC6426592

[29] Kim M, Lee Y, Park B, et al. Hepatitis virus B and C infections are associated with an increased risk of non‐Hodgkin lymphoma: a nested case‐control study using a national sample cohort. J Med Virol. 2020;92(8):1214–1220. 31825111 10.1002/jmv.25653

[30] Desai S, Fernandez S, Vakiti A, et al. Virologic clearance of hepatitis C is associated with improved outcomes in African American patients with lymphoma. Blood. 2019;134(Supplement_1):1614–1614.

[31] Smedby KE, Ponzoni M. The aetiology of B-cell lymphoid malignancies with a focus on chronic inflammation and infections. J Intern Med. 2017;282(5):360–370. 28875507 10.1111/joim.12684

[32] Cho SF, Wu WH, Yang YH, et al. Longitudinal risk of herpes zoster in patients with non-Hodgkin lymphoma receiving chemotherapy: a nationwide population-based study. Sci Rep. 2015;5:14008. 26391893 10.1038/srep14008 PMC4585724

[33] Gao P, Lazare C, Cao C, et al. Immune checkpoint inhibitors in the treatment of virus-associated cancers. J Hematol Oncol. 2019;12(1):58. 10.1186/s13045-019-0743-4 PMC6558794 31182108

[34] Welch H, Schwartz L, Woloshin S. Are increasing 5-YEAR survival rates evidence of success against cancer? JAMA 2000;283(22):2975–2978. 10865276 10.1001/jama.283.22.2975

